# Editorial: DNA damage response pathways meet endocrine systems

**DOI:** 10.3389/fendo.2023.1138326

**Published:** 2023-01-27

**Authors:** Haim Werner, Leonard Girnita

**Affiliations:** ^1^ Department of Human Molecular Genetics and Biochemistry, Sackler School of Medicine, Tel Aviv University, Tel Aviv, Israel; ^2^ Department of Oncology and Pathology, BioClinicum, Karolinska Institutet and Karolinska University Hospital, Stockholm, Sweden

**Keywords:** DNA damage (DDR), DNA repair, DNA methylation, Ewing family of tumors, mutation

The aim of the *DNA damage response pathways meet endocrine systems* RT is to provide a comprehensive, *state-of-the-art* overview of current trends and approaches in the analysis of DNA damage in the specific context of endocrinology. DNA damage affects all major cellular processes. Investigation of the impact of DNA damage on disease has dramatically expanded over the past few decades. The DNA damage response and DNA repair pathways were predominantly examined in the framework of cancer research. Lessons learned from the biology of DNA damage generated therapeutic opportunities that were successfully implemented in clinical oncology. Besides cancer, DNA damage is being also investigated in other biological areas, including aging, reproduction and the endocrine system. Dissecting the mechanisms that are responsible for the DNA damage response in endocrine systems is an important novel area in the field of endocrinology.

In an article entitled “*Characterization of the CpG island methylator phenotype subclass in papillary thyroid carcinoma*” Gu et al identified two distinct methylation subgroups among papillary thyroid carcinoma patients. Subgroups were termed: (i) the ‘CpG island methylator phenotype’ (CIMP) subgroup (elevated rates of DNA methylation); and (ii) nCIMP subgroup (reduced rates of DNA methylation). Authors investigated the impact of the different methylation patterns on patient’s prognosis, immune profiles and response to immunological therapy or chemotherapy. Authors suggest that ‘*CIMP might serve as a tool for risk stratification and help to make medication guide for patients of different subtypes’*.

In a paper entitled “*Integrative analysis reveals novel associations between DNA methylation and the serum metabolome of adolescents with type 2 diabetes: A cross- sectional study*” Agarwal et al conducted a cross-sectional investigation of DNA methylation in blood mononuclear cells from adolescents with type 2 diabetes with serum metabolomic data. The aim of the analysis was to explore modifications in the epigenetic landscape of white blood cells that might correlate with an affected serum metabolome. Authors identified differentially methylated regions in associations with a number of important metabolites in patients. Among others, metabolites include the upstream transcription factor-2 (USF2), free fatty acid receptor-1 (FFAR1), *etc*. Authors conclude that ‘*data may help generate new hypotheses to investigate the mechanisms that influence the metabolomic profile of adolescents diagnosed with T2D’*.

Mutations in the B-Raf proto-oncogene (BRAF) are associated with a variety of solid tumors, including papillary thyroid carcinoma. In a paper entitled “*A comparison of DP-TOF Mass Spectroscopy (MS) and Next Generation Sequencing (NGS) methods for detecting molecular mutations in thyroid nodules fine needle aspiration biopsies*” (Qian et al.) authors compared DP-TOF, a DNA MS platform, and NGS methods for detecting multiple gene mutations (including BRAFV600E) in aspiration fluid from 93 patients. Authors suggest that ‘*the MS method can be used as an inexpensive, accurate, and dependable initial screening method to detect genes mutations and as an adjunct to clinical diagnosis’*.

Ewing sarcoma (EWS) is a poorly differentiated tumor with characteristic reciprocal chromosomal translocation, involving the *EWS* gene and one of the members of the *ETS* gene family. EWS is the second most common tumor of soft tissue and bone in children and adolescents and has a high propensity for metastasis. Disruption of DNA methylation patterns was shown to impact EWS etiology. In an article entitled “*Novel targeting of DNA methyltransferase activity inhibits Ewing sarcoma cell proliferation and enhances tumor cell sensitivity to DNA damaging drugs by activating the DNA damage response*” Cristalli et al evaluate the efficacy of MC3343, a quinoline-based DNA methyltransferase (DNMT) inhibitor with high activity against cancer cells, including EWS. Authors provide evidence that MC3343 impairs DNMT1 expression but does not influence methylation of EWS cells. Authors conclude that ‘*MC3343 slowed proliferation, induced DNA damage and cell death, and acted synergistically with other DNA damaging drugs, thereby indicating its potential use as an interesting adjuvant therapeutic agent against EWS’*.

Polycystic ovary syndrome (PCOS) is a female endocrine disorder associated with poor pregnancy outcomes. It is unknown whether sperm quality affects pregnancy of PCOS patients. The consequences of oocyte *in vitro* fertilization (IVF) by sperm cells exhibiting DNA fragmentation is reported in a paper entitled “*The effect of sperm DNA fragmentation on in vitro fertilization outcomes for women with polycystic ovary syndrome*” (Wang et al.). Authors provide evidence that oocytes fertilized using sperm with a high DNA fragmentation index ‘*led to a lower high-quality blastocyst formation rate but had no influence on fertilization, high-quality embryo, clinical pregnancy and miscarriage rates’*.

The impact of female body mass index (BMI) on the DNA repair ability in oocytes after fertilization is unknown. Li et al evaluated the embryo quality and reproductive potential of oocytes from overweight women upon fertilization with sperm with different degrees of DNA fragmentation. The paper, entitled “*Decreased DNA repair ability: A mechanism for low early embryonic development potential of oocytes from overweight patients after fertilization in IVF cycles*”, is based on 1,612 patients undergoing fresh autologous IVF. Patients were divided into two categories according to their BMI (normal weight *versus* overweight). Groups were subdivided into two subgroups according to sperm DNA fragmentation index [low (LF) *versus* high (HF) fragmentation]. For the normal weight group, there were no differences in embryo quality and reproductive potential between the LF and HF groups. However, in the overweight group, the HF subgroup exhibited lower fertilization rates, blastocyst development rates and high-quality blastocyst rates, despite similar pregnancy rates. Authors conclude that ‘*decreased DNA repair activity in oocytes may be a possible mechanism for the low early development potential of embryos from overweight patients in IVF cycles’*.

The diagnosis of follicular thyroid carcinoma (FTC) is very complex given the fact that commonly used screening methods could not diagnose FTC preoperatively. Yao et al conducted an analysis of DNA methylation and RNA array data aimed at identifying thyroid cancer-specific DNA methylation markers. The article, entitled “*Integrative analysis of DNA methylation and gene expression identified follicular thyroid cancer-specific diagnostic biomarkers*”, was based on 14 FTC *versus* 16 benign thyroid lesions. Authors identified four DNA methylation sites that probably, might be used for screening and identification of FTC patients. Authors emphasize ‘*the potential use of methylation markers in FTC diagnosis’*.

In summary, the feedback and feedforward regulatory mechanisms linking the endocrine systems to the DNA damage response pathway play central roles in various human disorders. The collection of papers presented in this RT further advances the understanding of intricacies of this system and how cells, tissues and organs modulate their responses and preserve homeostasis in the face of ever-changing conditions. Given their importance, endocrine system/DNA damage response biology is an intensely studied field, nevertheless many fundamental questions remain unanswered. For example, how coding and non-coding RNAs responses are coordinated by the endocrine system to avoid DNA-damage stress, what underlies the tissue-specific DNA damage responses, and how do cells modify their responses during cell division. Seeking answers to these and other questions is a fundamental research field for the near future.

## Author contributions

All authors listed have made a substantial, direct, and intellectual contribution to the work and approved it for publication.

